# Predictors of opportunistic illnesses incidence in post combination antiretroviral therapy era in an urban cohort from Rio de Janeiro, Brazil

**DOI:** 10.1186/s12879-016-1462-x

**Published:** 2016-03-22

**Authors:** Lara E. Coelho, Sandra W. Cardoso, Rodrigo T. Amancio, Ronaldo I. Moreira, Sayonara R. Ribeiro, Alessandra B. Coelho, Dayse P. Campos, Valdiléa G. Veloso, Beatriz Grinsztejn, Paula M. Luz

**Affiliations:** Instituto de Nacional de Infectologia Evandro Chagas, Fundação Oswaldo Cruz, Avenida Brasil 4365, Manguinhos, CEP: 21045-900 Rio de Janeiro Brazil

**Keywords:** AIDS-related opportunistic infections, HIV, Acquired immunodeficiency syndrome, Incidence, Cohort study, Cox proportional hazards regression models

## Abstract

**Background:**

Opportunistic illnesses still account for a huge proportion of hospitalizations and deaths among HIV-infected patients in the post combination antiretroviral therapy (cART) era, particularly in middle- and low-income countries. The aim of this study was to assess predictors of the top four most incident opportunistic illnesses (tuberculosis, esophageal candidiasis, cerebral toxoplasmosis and *Pneumocystis jiroveci* pneumonia) in an HIV clinical cohort from a middle-income country in the post cART era.

**Methods:**

A total of 2835 HIV infected participants aged ≥ 18 years at enrollment were followed from January 2000 to December 2012 until the occurrence of their first opportunistic illness, death or end of study, whichever occurred first. Extended Cox proportional hazards regression models, stratified by use of cART, were fitted to assess predictors of opportunistic illness incidence during follow-up.

**Results:**

The incidence rates of tuberculosis, esophageal candidiasis, cerebral toxoplasmosis and *Pneumocystis jiroveci* pneumonia were 15.3, 8.6, 6.0, 4.8 per 1000 persons-year, respectively. Disease specific adjusted Cox models showed that presence of an opportunistic illness at enrollment significantly increased disease incidence while higher nadir CD4+ T lymphocyte count had a significant protective effect in patients not in use of cART. Duration of cART use also significantly reduced disease incidence.

**Conclusions:**

Our findings show that, still in the post-cART era, prevention of opportunistic infections can be achieved by preventing immune deterioration by instituting early use of cART. Interventions focusing on early diagnosis and linkage to care in addition to the prompt initiation of cART are essential to reduce the incidence of opportunistic illnesses among HIV infected patients in post-cART era.

## Background

Despite the virological control and immunological recovery achieved with the use of combination antiretroviral therapy (cART), opportunistic illnesses continue to occur at an unacceptable frequency and account for a huge proportion of hospitalizations and deaths in HIV infected patients in post-cART era [1–6]. Opportunistic illnesses occurrence remains mostly related to late presentation and linkage to care and to poor adherence [1, 4, 7]. Late diagnosis and difficulties in access to health care are a major challenge in the current HIV/AIDS epidemic, and both in high- and low/middle-income settings more than a third of HIV infected patients start treatment with advanced disease [8–13]. Furthermore, poor adherence to antiretroviral therapy and emergence of multidrug resistance are associated with the occurrence of opportunistic illnesses in cART experienced patients [14]. In this study, we assessed the predictors of the four most incident opportunistic illnesses in the post-cART era in an urban cohort from a middle-income country [15].

## Methods

Instituto Nacional de Infectologia Evandro Chagas (INI, formerly known as Instituto de Pesquisa Clínica Evandro Chagas/IPEC) is a reference center for care and treatment of HIV infected patients, in Rio de Janeiro, Brazil, since 1986. Patients included in the cohort follow Brazilian guidelines for HIV treatment and opportunistic infections prophylaxis [16]. Cohort procedures have been described elsewhere [3, 17]. For this study we included adult patients (age > =18 years) at cohort entry, who were followed for a period of at least 60 days from January 1st 2000 to December 31st 2012. The start of the observation period was defined based on the first medical appointment at INI. Follow-up ended at the date of the first opportunistic illness for those who developed an opportunistic illness during follow-up. For patients who never developed an opportunistic illness during follow-up, end of follow-up was defined as the date of death, date of last clinical visit or study closure, whichever occurred first. Patients whose date of last clinical visit was prior to January 1st 2012 were considered loss to follow-up. Exclusion criteria included: patients with reported intravenous drug use (*n* = 31 patients) and patients with no race nor educational level information (*n* = 32). Since the outcome of interest was the occurrence of the first opportunistic illness after cohort enrollment, diagnoses occurring up to 30 days after the date of cohort enrollment were considered prevalent cases, and thus excluded from the count of incident cases.

To explore which factors were associated with the incidence of the first opportunistic illness, we used extended Cox proportional hazards models accounting for competing risks. Specifically, we evaluated the predictors for the four most incident opportunistic illnesses in our cohort in post-cART era, namely tuberculosis, esophageal candidiasis, cerebral toxoplasmosis and *Pneumocystis jirovecci* pneumonia (PCP) [15]. Thus, the following endpoints were included in the model: incident cases of tuberculosis, esophageal candidiasis, cerebral toxoplasmosis, PCP, and other opportunistic illnesses (those included in Centers for Disease Control and Prevention/CDC 1993 definition), as well as death.

The independent variables included in the models were age, combined gender/mode of HIV acquisition (women vs. heterosexual men vs. men who have sex with men), race/ethnicity, educational level, presence of opportunistic illness at enrollment (with a +/−30 day time-frame from the enrollment date), nadir of CD4+ T lymphocyte count, CD4+ T lymphocyte count and HIV viral load before end of follow up (within the 6 months prior to), and cART use (defined as two or more nucleoside reverse transcriptase inhibitors plus a nonnucleoside reverse transcriptase inhibitor or a protease inhibitor).

Demographical and clinical characteristics were compared between groups using Kruskal-Wallis test for continuous variables and chi-squared for categorical variables. Nadir CD4+ T lymphocyte count was unknown for 14 patients, the median of nadir CD4+ T lymphocyte count distribution was used as the imputed value. Kaplan-Meier survival curves, stratified by cART use, were plotted to show how the survival experience varied by type of event. Adjusted extended Cox proportional hazards regression models included all demographic and clinical variables explored in the unadjusted models (except for last CD4+ T lymphocyte count and last HIV viral load), since all constitute potential confounding variables.

We tested the proportional hazards assumption for each variable and globally in all final models using Schoenfeld Residuals. We found that the proportional hazards assumption was violated for the variable cART use. To address this model finding, we stratified patient’s follow up time into pre- and post-cART initiation and explored predictors of the four most incident opportunistic illnesses for the two scenarios: when cART was not used (without cART use models) and when cART was used (with cART use models). All models were again tested for the proportional hazards assumption and found to not violate this assumption. In an additional analysis, we explored the effect of last CD4+ T lymphocyte count and last HIV viral load in the models that considered those who used cART. Three hundred and eighty four patients did not have either information. In order to not exclude them from the analysis, which have resulted in a significant decrease in the number of events from 60 to 39, we imputed values. For those missing the last CD4+ T lymphocyte, the median of the last CD4+ T lymphocyte count distribution was used, and for those missing the last HIV viral load a random imputation of less than 400 copies/mm^3^ or 400 or more copies/mm^3^ was performed. R software (version 3.0.3), survival and mstate libraries were used for survival models analysis.

This study was approved by the ethics committee of INI (CAAE 0032.0.009.000–10) and was conducted according to the principles expressed in the Declaration of Helsinki. All patient records/information was de-identified prior to analysis. Participants provided written informed consent.

## Results

Overall, 2898 HIV infected patients aged 18 years or more enrolled the INI cohort between January 1st 2000 and November 1st 2012 and were followed-up for at least 60 days. After excluding 31 patients who reported intravenous drug use and 32 patients with no race nor educational level information, 2835 patients were included in the present study, 566 of which developed an opportunistic illness during follow-up (Table 1). The study population accounted for total follow-up of 11,571 PY (mean follow-up of 4.1 years per patient). Ninety-one (3.2 %) individuals were deemed loss to follow-up, yielding a rate of loss to follow-up of 7.9/1000PY.

**Table 1:** Demographic and clinical characteristics of the patients in the cohort and stratified by the occurrence of specific opportunistic illnesses

	Tuberculosis	Esophageal candidiasis	Cerebral toxoplasmosis	PCP	All patients^a^	
	*N* = 177	*N* = 100	*N* = 70	*N* = 55	*N* = 2835	*p*-value
Incidence rate^b^ (CI 95 %)	15.3 (13.2, 17.7)	8.6 (7.1, 10.5)	6.0 (4.8, 7.6)	4.8 (367, 6.2)	–	<0.001
Age, median (IQR)	35.4 (28.4,41.5)	38.6 (32.6,45)	33.8 (28.1,40.9)	39.1 (29.6,45.1)	35.6 (28.7,42.7)	0.02
18–29 years (%)	50 (28.2)	15 (15)	21 (30)	13 (23.6)	750 (26.5)	0.05
30–39 years (%)	67 (37.9)	36 (36)	27 (38.6)	13 (23.6)	1035 (36.5)	
40–49 years (%)	46 (26)	34 (34)	16 (22.9)	19 (34.5)	731 (25.8)	
50+ years (%)	14 (7.9)	15 (15)	6 (8.6)	10 (18.2)	319 (11.3)	
Gender-risk						0.21
Women	57 (32.2)	41 (41)	27 (38.6)	11 (20)	930 (32.8)	
Heterossexual men	59 (33.3)	27 (27)	23 (32.9)	22 (40)	833 (29.4)	
MSM	61 (34.5)	32 (32)	20 (28.6)	22 (40)	1072 (37.8)	
Race/ethnicity						0.12
White (%)	73 (41.2)	40 (40)	29 (41.4)	32 (58.2)	1415 (49.9)	
Non-white (%)	104 (58.8)	60 (60)	41 (58.6)	23 (41.8)	1420 (50.1)	
Educational level						0.03
0–8 years (%)	117 (66.1)	66 (66)	48 (68.6)	25 (45.5)	1399 (49.3)	
9+ years (%)	60 (33.9)	34 (34)	22 (31.4)	30 (54.5)	1436 (50.7)	
Nadir CD4 T-cell count (cells/mm^3^)						
Median (IQR)	107 (48,218)	74.5 (21,188.2)	63.5 (20,159.5)	55 (12,146)	196 (71,311.5)	0.002
<50 (%)	45 (25.4)	39 (39)	33 (47.1)	25 (45.5)	549 (19.4)	0.03
50–199 (%)	82 (46.3)	39 (39)	27 (38.6)	22 (40)	898 (31.7)	
200–349 (%)	40 (22.6)	17 (17)	8 (11.4)	5 (9.1)	847 (29.9)	
350+ (%)	10 (5.6)	5 (5)	2 (2.9)	3 (5.5)	541 (19.1)	
Opportunistic illness at enrolmment	44 (24.9)	43 (43)	35 (50)	21 (38.2)	211 (7.4)	<0.001
cART use during follow-up (%)	30 (16.9)	10 (10)	12 (17.1)	8 (14.5)	1878 (66.2)	0.43
Time of cART use in years^c^, median (IQR)	1.1 (0.6,2.7)	0.8 (0.4,1.3)	0.6 (0.1,1.6)	1.7 (1.1,2.1)	2.2 (0.9,4.1)	0.32

*cART* combination antiretroviral therapy, *PCP Pneumocystis jiroveci* pneumonia, *HR*, hazard ratio, *CI* confidence interval, *MSM* men who have sex with men

^a^Includes the entire study population

^b^Per 1000 person-years

^c^For those who used cART before end of follow-up

Tuberculosis (incidence rate [IR]: 15.3/1000 person-years [PY]), esophageal candidiasis (IR: 8.6/1000PY), cerebral toxoplasmosis (IR: 6.0/1000PY) and PCP (4.8/1000PY) were the four most incident illnesses in the period. In total, cases of tuberculosis, esophageal candidiasis, cerebral toxoplasmosis and PCP accounted for 71 % (402 out of 566) of the diseases. The median age at cohort enrollment was 35.6 years (interquartile range [IQR]: 28.7–42.7). There was a significant difference in the age at cohort enrollment among the groups of patients who developed each of the four diseases, with older patients in the PCP group (18 % of patients with 50 years or more) and a younger range of patients in the tuberculosis and the cerebral toxoplasmosis groups. One third of the cohort patients were women, the highest proportion of women was seen in the esophageal candidiasis group (41 %) in contrast with the smallest proportion of women observed in PCP group (20 %). Almost half of the patients were non-white and had less than 9 years of formal education (Table 1).

Overall, the median nadir CD4+ T lymphocyte count of the cohort was low (196 cells/mm^3^, IQR: 71–312). When comparing the groups of patients who developed the opportunistic illnesses of interest we found that patients who developed PCP had the lowest nadir CD4+ T lymphocyte counts (median of 55 cells/mm^3^ [IQR: 12–146], with 46 % of the patients having less than 50 cells/mm^3^), followed closely by those who developed cerebral toxoplasmosis (median of 63 cells/mm^3^ [IQR: 20–160], with 47 % of the patients having less than 50 cells/mm^3^). Patients who developed tuberculosis had the highest nadir CD4+ T lymphocyte (median of 107 cells/mm3, IQR: 48–218). Overall, 66 % of the patients used cART before end of follow-up. Compared to the entire cohort, the use of cART was less frequent among patients that developed one of the four studied diseases. Although not significantly different, the esophageal candidiasis group had the smallest proportion of cART users (10 %) while the tuberculosis and cerebral toxoplasmosis groups had 17 % of cART users. As for the duration of cART use there was no significant difference among the groups, with the shortest time for the cerebral toxoplasmosis group (median: 0.6 year, IQR:0.1, 1.6) and the longest time for PCP (median: 1.7 year, IQR: 1.1, 2.1) (Table 1).

**Fig. 1:**
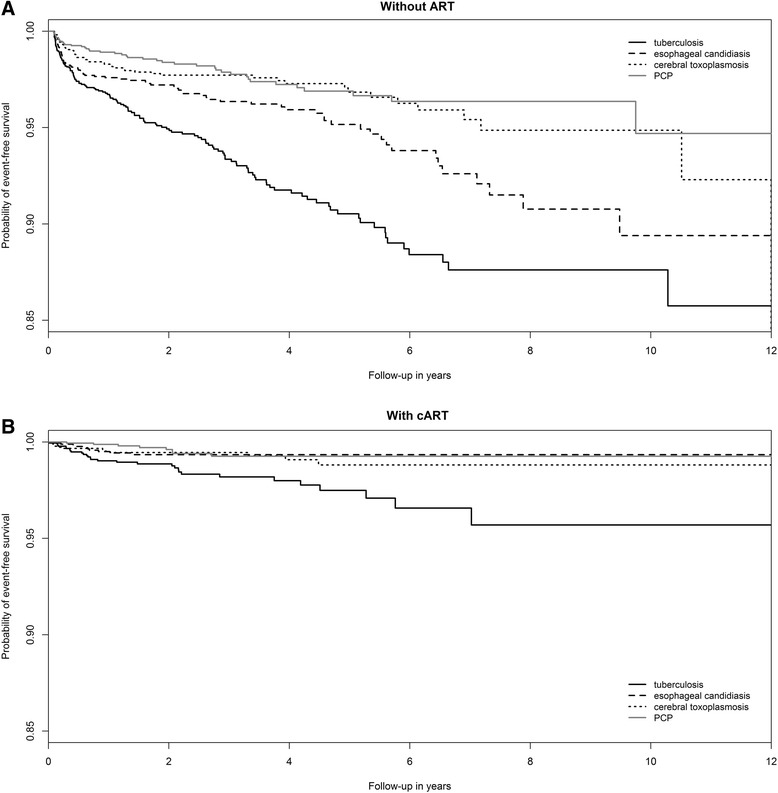
Kaplan-Meier survival curves illustrating the probability of event-free survival over follow-up time for patients who never used cART (*top*) and for those who used cART (*bottom*)

The 2835 included patients accounted for 6509 PY not on cART and 5062 PY on cART. The Kaplan-Meier survival curves illustrating the probability of event-free survival over follow-up time without cART (Fig. 1a) and with cART (Fig. 1b) shows that the incidence of all four diseases occurs more abruptly without cART than with cART. The incidence rate of opportunistic illnesses was significantly lower when cART was used compared to when cART was not used (73.6/1000 PY without cART vs. 17.2/1000 PY with cART, *p* < 0.001).

In the non-cART models, non-white race/ethnicity (hazard ratio [HR] 1.47, 95 % confidence interval [95 % CI] 1.04–2.06) and lower educational level (<9 years of formal education) (HR 1.64, 95 % CI 1.14–2.35) were associated with an increased hazard of tuberculosis. Higher nadir CD4+ T lymphocyte count (per 100 cells/mm^3^) was associated with significantly reduced hazard of tuberculosis (HR 0.79, 95 % CI 0.70–0.89), esophageal candidiasis (HR 0.74, 95 % CI 0.62–0.89), cerebral toxoplasmosis (HR 0.62, 95 % CI 0.48–0.81) and PCP (HR 0.58, 95 % CI 0.43–0.79). The presence of an opportunistic illness at enrollment was also significantly associated with an increased hazard for all four studied illnesses (Table 2).

**Table 2:** Adjusted Cox hazards regression models for specific opportunistic illnesses for patients who did not use combination antiretroviral therapy (*n* = 2750 individuals)

	Tuberculosis	Esophageal candidiasis	Cerebral Toxoplamosis	PCP
	(*N* = 147)	(*N* = 90)	(*N* = 58)	(*N* = 47)
	HR (CI)	HR (CI)	HR (CI)	HR (CI)
Gender-Risk				
Women	Ref.	Ref.	Ref.	Ref.
Heterosexual men	1.29 (0.85–1.95)	0.71 (0.43–1.19)	0.67 (0.35–1.26)	2.10 (0.91–4.84)
MSM	1.45 (0.95–2.20)	1.02 (0.61–1.71)	0.89 (0.46–1.71)	1.97 (0.84–4.64)
Age (per year)	0.98 (0.96–1.00)	**1.03 (1.00–1.04)**	1.00 (0.97–1.03)	1.01 (0.98–1.04)
Race/ethnicity				
White	Ref.	Ref.	Ref.	Ref.
Non-white	**1.47 (1.04–2.06)**	1.29 (0.83–2.00)	1.40 (0.80–2.45)	0.71 (0.39–1.31)
Educational level				
0–8 years	**1.64 (1.14–2.35)**	1.41 (0.88–2.26)	1.39 (0.77–2.53)	0.71 (0.38–1.33)
9+ years	Ref.	Ref.	Ref.	Ref.
Nadir CD4+ T lymphocyte (per 100 cells/mm^3^)	**0.79 (0.70–0.89)**	**0.74 (0.62–0.89)**	**0.62 (0.48–0.81)**	**0.58 (0.43–0.79)**
Opportunistic illness at enrollment	**1.61 (1.04–2.52)**	**6.27 (3.94–9.96)**	**5.65 (3.23–9.89)**	**4.10 (2.15–7.82)**

*PCP Pneumocystis jiroveci* pneumonia, *HR* hazard ratio, *CI* 95 % confidence interval, *MSM* men who have sex with men

Bold font implies statistically significant results assuming a 5 % significance threshold

In the cART use models, the above described associations of non-white race and lower educational level with increased hazard of tuberculosis were no longer observed. In contrast, the presence of an opportunistic illness at enrollment still led to a significantly increased hazard, even stronger than that observed in non-cART models, for all studied diseases. Additionally, per year use of cART significantly reduced the hazard of tuberculosis (HR 0.78, 95 % CI 0.63–0.97) and cerebral toxoplasmosis (HR 0.61, 95 % CI 0.39–0.96). For esophageal candidiasis and PCP, although we had found a protective effect of per year use of cART, the estimated effects did not reach statistical significance (Table 3).

**Table 3:** Adjusted Cox hazards regression models for specific opportunistic illnesses for patients who used combination antiretroviral therapy (*N* = 1878 individuals)

	Tuberculosis	Esophageal candidiasis	Cerebral Toxoplamosis	PCP
	(*N* = 30)	(*N* = 10)	(*N* = 12)	(*N* = 8)
	HR (CI)	HR (CI)	HR (CI)	HR (CI)
Gender-Risk				
Women	Ref.	Ref.	Ref.	Ref.
Heterosexual men	0.85 (0.38–1.88)	0.15 (0.02–1.25)	1.46 (0.39–5.43)	0.81 (0.16–4.20)
MSM	0.53 (1.89–1.58)	0.25 (0.05–1.42)	0.31 (0.05–2.00)	0.59 (0.08–4.16)
Age (per year)	1.02 (0.99–1.06)	0.93 (0.86–1.02)	**0.91 (0.84–0.99)**	1.07 (1.00–1.14)
Race/ethnicity				
White	Ref.	Ref.	Ref.	Ref.
Non-white	0.86 (0.39–1.88)	2.10 (0.43–10.29)	0.46 (0.14–1.51)	1.26 (0.27–5.87)
Educational level				
0–8 years	2.08 (0.78–5.51)	0.89 (0.19–4.12)	1.22 (0.29–5.10)	0.38 (0.07–2.03)
9+ years	Ref.	Ref.	Ref.	Ref.
Nadir CD4+ T lymphocyte (per 100 cells/mm^3^)	0.85 (0.63–1.15)	0.73 (0.42–1.25)	0.89 (0.59–1.33)	1.25 (0.83–1.90)
Opportunistic illness at enrollment	**23.0 (9.60–55.2)**	**13.6 (2.93–63.3)**	**34.6 (8.50–141.4)**	**21.5 (2.86–162.1)**
Time under cART (per year)	**0.78 (0.63–0.97)**	0.55 (0.29–1.03)	**0.61 (0.39–0.96)**	0.60 (0.33–1.08)

PCP *Pneumocystis jiroveci* pneumonia, *HR* hazard ratio, *CI* 95 % confidence interval, *MSM* men who have sex with men

Bold font implies statistically significant results assuming a 5 % significance threshold

The additional analysis showed a significant protective effect of higher last CD4+ T lymphocyte count on the incidence of cerebral toxoplasmosis (HR 0.70 per 100 cell/mm^3^, 95 % CI 0.49–0.99, Table 4). For all other illnesses, neither last CD4+ T lymphocyte count nor last HIV viral load were significantly associated with the incidence of opportunistic illnesses. Of note is the fact that the addition of these variables to the model presented in Table 3 did not significantly change the effect of the other variables.

**Table 4:** Adjusted Cox hazards regression models (including last CD4 and last viral load as independent variables) for patients who used antiretroviral therapy (N = 1878 individuals)

	Tuberculosis	Esophageal candidiasis	Cerebral Toxoplamosis	PCP
	(*N* = 30)	(*N* = 10)	(*N* = 12)	(*N* = 8)
	HR (CI)	HR (CI)	HR (CI)	HR (CI)
Gender-Risk				
Women	Ref.	Ref.	Ref.	Ref.
Heterosexual men	0.82 (0.37–1.82)	0.16 (0.02–1.39)	1.10 (0.27–4.43)	0.90 (0.17–4.73)
MSM	0.49 (0.16–1.48)	0.37 (0.06–2.24)	0.34 (0.04–2.71)	0.57 (0.08–4.09)
Age (per year)	1.02 (0.98–1.06)	0.94 (0.86–1.02)	**0.90 (0.82–0.99)**	1.07 (1.00–1.14)
Race/ethnicity				
White	Ref.	Ref.	Ref.	Ref.
Non-white	0.90 (0.41–2.00)	1.84 (0.35–9.51)	0.53 (0.14–1.92)	1.05 (0.21–5.19)
Educational level				
0–8 years	1.89 (0.70–5.12)	1.05 (0.22–5.12)	1.46 (0.34–6.33)	0.44 (0.08–2.41)
9+ years	Ref.	Ref.	Ref.	Ref.
Nadir CD4+ T lymphocyte (per 100 cells/mm^3^)	0.88 (0.62–1.24)	0.95 (0.50–1.79)	1.17 (0.85–1.61)	1.15 (0.73–1.83)
Last CD4+ Tlymphocyteª (per/100 cells/mm^3^)	0.95 (0.80–1.13)	0.87 (0.61–1.23)	**0.70 (0.49–0.99)**	1.09 (0.90–1.33)
Last HIV viral load^b^				
<400 copies/mm^3^	Ref.	Ref.	Ref.	Ref.
>400 copies/mm^3^	0.35 (0.08–1.56)	4.45 (0.98–20.19)	3.04 (0.73–12.58)	2.57 (0.42–15.88)
Opportunistic illness at enrollment	**23.01 (9.44–56.09)**	**15.52 (3.38–71.30)**	**31.96 (8.31–123.88)**	**21.83 (2.85–167.29)**
Time under cART (per year)	**0.79 (0.63–0.99)**	0.71 (0.40–1.28)	0.70 (0.44–1.12)	0.60 (0.33–1.08)

*cART* combination antiretroviral therapy, *PCP Pneumocystis jiroveci* pneumonia, *HR* hazard ratio, *CI* 95 % confidence interval, *MSM* men who have sex with men

ªData imputed for 333 individuals with missing last CD4

^b^Data imputed for 368 individuals with missing last HIV viral load

Bold font implies statistically significant results assuming a 5 % significance threshold

## Discussion

In this analysis, we studied the predictors of the four most incident opportunistic illnesses in the post-cART era in an urban cohort of HIV-infected individuals from Rio de Janeiro, Brazil. In the non-cART models, we showed a significant protective effect of higher nadir CD4+ T lymphocyte count in reducing the incidence all illnesses. When modeling the predictors of incident opportunistic illnesses in the presence of cART, increased duration of cART led to a decreased incidence of all studied illnesses. Additionally, in the models with and without cART, we found that the presence of an opportunistic illness at cohort enrollment was strongly associated with an increased incidence of all studied illnesses.

We found that tuberculosis was the most incident opportunistic illness, almost twice as frequent as the second most frequent disease (incidence rates of 15.3/1000PY vs. 8.6/1000PY of tuberculosis and esophageal candidiasis, respectively, during the study period). In countries with a high tuberculosis burden, as is the case for Brazil, Rio de Janeiro in particular, HIV and tuberculosis co-infection represent a huge public health challenge; tuberculosis accounts for the majority of hospitalizations and deaths in HIV infected populations [5, 18–20]. In the absence of cART, we observed that proxies of poverty (i.e. non-white race/ethnicity and low education) led to higher tuberculosis incidence, as it occurs in the general population [21, 22]. Our findings thus likely reflect the local high burden of tuberculosis and shows how it leads to increased morbidity also for HIV infected individuals. Additionally, and differently from other opportunistic illnesses, tuberculosis can occur in HIV-infected patients who may not have severe immunodeficiency, a finding also shown in our study by the higher median nadir CD4+ T lymphocyte count among those who developed tuberculosis compared to those who developed other diseases. Nonetheless, similarly to data already published [19, 23], we demonstrated that for patients not using cART, higher nadir CD4+ T lymphocyte counts significantly reduced the incidence of tuberculosis. Moreover, for patients who developed tuberculosis while in use of cART, we observed a per year increased protection of cART use, while controlling for CD4+ T lymphocyte count. This finding has been described in other settings with a high burden tuberculosis and indicates that the early use of cART can promote recovery of mycobacterial-specific immunity reducing the risk of active tuberculosis [24–26]. It could be argued that for patients using cART, the occurrence of opportunistic illnesses can be related to inflammatory reconstitution of the immune system (IRIS) and unmasking of previous opportunistic illnesses, this being particularly relevant for tuberculosis [27]. However, for our results this hypothesis loses strain when we observe that the lower limit of the interquartile range of duration of cART use among patients who developed tuberculosis was superior to 6 months, with median of 1.1 year, which speaks against IRIS and unmasking tuberculosis in our study.

In this study, we also explored the predictors of esophageal candidiasis, cerebral toxoplasmosis and PCP incidence and found that higher nadir CD4+ T lymphocyte count was highly protective among patients not under cART. Additionally, in the models with cART, duration of cART also decreased the incidence of all diseases. The strong effect of the presence of an opportunistic illness at enrollment in the risk of an incident opportunistic illness during follow-up corroborates the vast literature attesting to the benefits of preventing immune deteriorating with cART use [28–30] while also providing an updated analysis of the predictors of incidence of important opportunistic illnesses in a middle-income country with universal use of cART.

Recently, data published from several trials showed important results addressing early start of cART that should modify the standard of care of HIV infected patients [24, 31, 32]. They all reported strong evidence that early initiation of cART significantly reduces the risk of death or AIDS-defining illness irrespective of the CD4+ T lymphocyte count. In this study, we observed that the use of cART was associated with a 77 % incidence reduction and that for those patients not on cART, most opportunistic illnesses occurred early in the follow-up (less than 2 years). The results of the present study and the findings from the recent publications collectively suggest that the prompt start of cART after cohort enrollment could have prevented a signficant fraction of the opportunistic illnesses. It is therefore most fortunate that, since 2013, Brazilian guidelines have been updated and cART is now recommended for all HIV-infected individuals [16].

On the other hand, our results showed that 80 % of the patients had a nadir CD4+ T lymphocyte count under 350 cells/mm^3^, and that low nadir CD4+ T lymphocyte counts was associated with increased hazard of opportunistic illnesses. Late HIV diagnosis and late presentation to care persist as major challenges in Brazil and other median/low income settings [33]. A recent meta-analysis showed that one third of the HIV-infected population worldwide discovered their HIV status upon hospital admission [2]. So while early start of cART is a protective action to prevent AIDS progression, testing and linkage to care are essential to allow HIV-infected patients to benefit from this intervention.

We showed that there was a significant decrease in the incidence rates of opportunistic illnesses when cART was used compared to when it was not. However, our findings also show that despite this significant decrease in incidence, opportunistic illnesses continue to occur in cART experienced patients. We hypothesize that the reasons for clinical failure and disease progression among cART experienced patients likely include poor adherence, suboptimal response to cART and virological failure as other studies have shown [1, 7].

There are limitations to our study that should be acknowledged. First, we could not take into account the use of prophylactic isoniazid. Isoniazid prophylaxis is recommended by the World Health Organization in settings with a high burden of tuberculosis [34]. Studies have shown that isoniazid prophylaxis prevents tuberculosis [35, 36] particularly among those with positive tuberculin skin test (TST) [37, 38], and that this effect extends to HIV infected patients on cART, providing a long term protective effect [39–41]. Brazilian guidelines recommend the use of prophylactic isoniazid for HIV infected patients with reactive TST [42]. Despite formal recommendations, both TST and prophylactic isoniazid are still inconsistently used in Brazil, with a fraction of patients not being prescribed prophylactic isoniazid despite having a positive TST [43]. This inconsistent use limited our ability to explore the impact of prophylactic isoniazid on the incidence of tuberculosis, an important limitation of the present study. In fact, this limitation extends to the use of other prophylaxis such as for PCP and cerebral toxoplasmosis. To correctly account for prophylaxis use we would need detailed time dependent CD4+ T lymphocyte counts to allow us to determine who, when and for how long prophylaxis should haven prescribed and, among those, who in fact used it. Without this information, we cannot correctly compare the groups of patients that used prophylaxis as needed, to those that did not use despite the need, to those who never needed prophylaxis. Second, the lack of consistent CD4+ T lymphocyte counts at the moment of the diagnosis of the opportunistic illnesses led us use nadir CD4+ T lymphocyte count as a proxy of the patients’ immune status. To explore the impact of the last CD4+ T lymphocyte counts and last HIV viral load on opportunistic illnesses incidence we conducted an additional analysis among patients who used cART and found that they did not provide additional information while also not modifying the effects of the variables already explored. Third, we lacked information on adherence to cART, thus prescribed cART was considered in use until end of follow-up. It is likely that if adherence information was present to modulate the effect of cART an even stronger effect would have been seen for this variable and as such our results can be considered conservative estimates. Finally, our cohort population is non-probabilistic such that our findings may lack generalizability. Despite these limitations, our clinical care setting has a well-established and continuously updated database that provides reliable information on clinical events and deaths. Also, our setting provides a primary and specialty care including emergency assistance such that the patients who care at INI resort to it in case of illnesses making clinical events underestimation very unlikely.

## Conclusion

In summary, we have shown that, in post-cART era, prevention of opportunistic illnesses can be achieved by preventing immune deterioration by instituting early use of cART. Early HIV diagnosis and linkage to care are important challenges for HIV primary care, without which HIV-infected patients will not have the opportunity to benefit from the protective effects of cART. Efforts to diagnose and link HIV infected patients to primary care need to be intensified in order to diminish HIV associated morbidity. Early initiation of cART has been proved to be a protective intervention against AIDS progression and death, our results corroborate with these findings and reinforce the most recent position of the Brazilian Heath Ministry that advocates in favor of the test and treat strategy.

## Abbreviations

cART: combination antiretroviral therapy
CDC: Centers for Disease Control and Prevention
CI: confidence interval
HR: hazard ratio
INI: Instituto Nacional de Infectologia Evandro Chagas
IPEC: Insituto de Pesquisa Clínica Evandro Chagas
IRIS: inflammatory reconstitution of the immune system
MSM: men who have sex with men
PCP: *Pneumocystis jiroveci* pneumonia
PY: person-years.
TST: tuberculin skin test
